# Molecular modelling evaluation of exon 18 His845_Asn848delinsPro PDGFRα mutation in a metastatic GIST patient responding to imatinib

**DOI:** 10.1038/s41598-018-38028-x

**Published:** 2019-02-18

**Authors:** Margherita Nannini, Giuseppe Tarantino, Valentina Indio, Gloria Ravegnini, Annalisa Astolfi, Milena Urbini, Antonio De Leo, Donatella Santini, Claudio Ceccarelli, Elisa Gruppioni, Annalisa Altimari, Paolo Castellucci, Stefano Fanti, Valerio Di Scioscio, Maristella Saponara, Lidia Gatto, Andrea Pession, Pier Luigi Martelli, Rita Casadio, Maria Abbondanza Pantaleo

**Affiliations:** 1 Department of Specialized, Experimental and Diagnostic Medicine, Sant’Orsola-Malpighi Hospital, University of Bologna, Via Massarenti 9, 40138 Bologna, Italy; 2 “Giorgio Prodi” Cancer Research Centre, University of Bologna, Bologna, Italy; 3 Department of Pharmacy and Biotechnology, Via Irnerio 48, 40126 Bologna, Italy; 4 Pathology Unit, S.Orsola-Malpighi Hospital, Via Massarenti 9, 40138 Bologna, Italy; 5 Laboratory of Oncologic Molecular Pathology, S.Orsola-Malpighi Hospital, Via Massarenti 9, 40138 Bologna, Italy; 6 Nuclear Medicine Unit, University of Bologna, S. Orsola-Malpighi Hospital, Bologna, Italy; 7 Department of Radiology, S. Orsola Malpighi Hospital, University of Bologna, Bologna, Italy; 8 Biocomputing Group, Department of Pharmacy and Biotechnology, University of Bologna, Bologna, Italy

## Abstract

Platelet-Derived Growth Factor Receptor Alpha (PDGFRA) mutations occur in approximately 5–7% of gastrointestinal stromal tumours (GIST). Over half of all PDGFRA mutations are represented by the substitution at position 842 in the A-loop of an aspartic acid (D) with a valine (V), recognized as D842V, conferring primary resistance to imatinib *in vitro* and in clinical observations due to the conformation of the kinase domain, which negatively affects imatinib binding. The lack of interaction between imatinib and the D842V PDGFRA mutated model has been established and widely confirmed *in vivo*. However, for the other PDGFRA mutations, the correlation between pre-clinical and clinical data is still unclear. An *in silico* evaluation of the p.His845_Asn848delinsPro mutation involving exon 18 of PDGFRA in a metastatic GIST patient responding to first-line imatinib has been provided. Docking analyses were performed, and the ligand-receptor interactions were evaluated with the jCE algorithm for structural alignment. The docking simulation and structural superimposition analysis show that PDGFRA p.His845_Asn848delinsPro stabilizes the imatinib binding site with the residues that are conserved in KIT. The *in vivo* evidence that PDGFRA p.His845_Asn848delinsPro is sensitive to imatinib was confirmed by the molecular modelling, which may represent a reliable tool for the prediction of clinical outcomes and treatment selection in GIST, especially for rare mutations.

## Introduction

Platelet-Derived Growth Factor Receptor Alpha (PDGFRA) mutations are, by far, the most infrequent mutations of the two known driving kinase genes (KIT - Proto-Oncogene Receptor Tyrosine Kinase and PDGFRA) in gastrointestinal stromal tumours (GIST), and they occur in approximately 5–7% of cases^1,2^. PDGFRA belongs to the type III tyrosine kinase (TK) receptor family. This family is characterized by a specific molecular structure comprising an extracellular (EC) domain and a cytoplasmic domain with a juxtamembrane (JM) region and a TK domain. The EC and cytoplasmatic domain are connected by a transmembrane region. The activation of the receptor occurs as a result of the binding of ligands in the EC domain that lead to dimerization and to a phosphorylation cascade of tyrosine residues in multiple downstream signalling molecules. Inside the TK domain an activation loop (A-loop) has been described, and it conformationally regulates the ATP-binding pocket and leads to kinase activation.

Oncogenic PDGFRA mutations activate receptor TKs, resulting in a constitutive phosphorylation. Mutations in the EC domain lead to spontaneous receptor dimerization. Mutations in the cytoplasmic domain instead mainly affect the A-loop encoded by exon 18 (~5%), or more rarely the JM domain encoded by exon 12 (~1%), or the ATP binding domain encoded by exon 14 (<1%)^2^. PDGFRA, as well as the KIT receptor, can acquire two different conformations: active and auto-inhibited. The auto-inhibited form of KIT and PDGFRA are stabilized by the JM domain, which shields the kinase active site. Mutations in the JM domain affect its autoregulatory function and promote spontaneous kinase activation.

A crystallized structure of KIT in complex with imatinib demonstrates that inhibitor binding disrupts this natural mechanism for maintaining the auto-inhibited state of the JM domain, therefore inhibiting the enzyme activity of the protein semicompetitively. In the auto-inhibited PDGFRA kinase structure there is an additional helix that orients the conserved residues of Asp836-Phe837-Gly838 (DFG) of the A-loop in a “DFG out” conformation^3^.

Over half of all PDGFRA mutations are represented by the substitution at position 842 in the A-loop of an aspartic acid (D) with a valine (V), recognized as D842V, conferring primary resistance to imatinib *in vitro* as well as in clinical observations due to the conformation of the kinase domain, which negatively affects imatinib binding^2,4–8^. Indeed, it was recently shown that modifications of the D842 residue interfere with a swinging movement of the activation loop, leading to a conformational shift of the ATP binding pocket from an active to an inactive conformation. The substitutions of aspartic acid with a valine also reduce the accessibility of the ATP pocket, contributing to resistance to the drug^9^.

Therefore, both the location and the nature of the mutation affect the affinity or binding of imatinib to the kinase and thus are important for imatinib sensitivity^2,7,8,10,11^.

As known, *in silico* studies of the receptor protein kinase are useful for predicting structural changes introduced by mutations and for predicting with the docking procedure the strength of the interactions between the protein model and the drug^12^.

The lack of interaction between imatinib and the D842V PDGFRA mutated model has been established and widely confirmed *in vivo*, considering also that PDGFRA is an integral cytoplasmic membrane protein. This imply several limitations including the process of crystallization, making fundamental the molecular modelling exploration. However, for the other PDGFRA mutations the correlation between pre-clinical and clinical data is still unclear. Herein, we provide an *in silico* evaluation of the p.His845_Asn848delinsPro mutation involving exon 18 of PDGFRA in a metastatic GIST patient responding to first-line imatinib.

## Methods

This study was approved by the local institutional ethical committee of Azienda Ospedaliero-Universitaria Policlinico S.Orsola-Malpighi (approval number 113/2008/U/Tess, 30 September 2008 approval code, approval date). Moreover, all methods were performed in accordance with the relevant guideline and regulations.

### Patient clinical history

**Figure 1:**
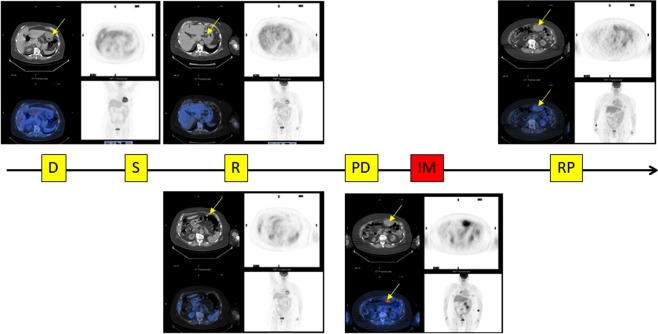
The patient’s clinical history organized as a timeline (D = diagnosis; S = surgery; R = relapse; PD = disease progression after stop IM for acute renal failure; IM = imatinib; RP = response after one month of IM).

The patient clinical history is reported in a simplified timetable, showing the more relevant clinical events (Fig. 1). Informed consent for study participation has been obtained from the patient.

**Figure 2:**
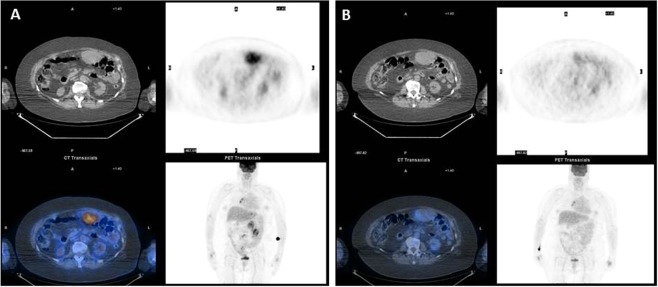
(**A**) The basal functional imaging by FDG-PET before starting imatinib revealed a pathological FDG up-take corresponding with several abdominal lesions. (**B**) Functional imaging by FDG-PET after one month of treatment revealing a complete metabolic response of all lesions.

Briefly, on June 2015, a 65-year-old female patient presenting with a gastric GIST 66 × 46 mm in diameter, Fluorodeoxyglucose Positron Emission Tomography (FDG-PET) scan negative, underwent a laparoscopic sub-total gastrectomy. The histological examination revealed a low-risk GIST according to Miettinen’s classification (site: stomach; size: 70 mm; mitotic index 4/50 high power field – HPF) harbouring an exon 18 mutation of PDGFRA. The surveillance programme was negative until November 2016, when a CT-scan showed multiple omental nodular lesions, FDG-PET negative, suggestive for GIST relapse. Given the unusual behaviour for a low-risk PDGFRA mutated GIST, a liver biopsy was suggested; however, the patient refused it. Therefore, the patient started a first-line treatment with imatinib at the standard dose of 400 mg daily, soon discontinued due to the onset of acute renal failure. In June 2017, the CT-scan evaluation showed disease progression, with an FDG-uptake in almost all lesions. Therefore, imatinib treatment at a lower dose of 200 mg was resumed, and the early FDG-PET evaluation after only one month of treatment showed a complete metabolic response of all lesions (Fig. 2). At the last follow-up control the patient was alive and still in treatment with a stable disease.

### Tumour characterization

#### Immunohistochemistry

The immunohistochemical analyses for CD117, PDGFRA and DOG1 were performed on 3 µm paraffin-embedded tumour sections using monoclonal pre-diluted anti-CD117 clone YR145 and anti-DOG-1 clone SP31 (Ventana Medical Systems, USA) or polyclonal anti-PDGFRα (Santa Cruz Biotech, USA) diluted 1:130 on Ventana Benchmark Ultra platform. Antigen Retrieval was performed in UltraCC1 Tris-HCl buffer pH 8,2-8,5 at 95 °C for 24–48 min, and the immunologic reaction was visualized with the OptiView DAB Detection Kit (Ventana, USA).

#### Molecular analysis

Mutational analyses of KIT (exons 11, 9, 13, 14 and 17) and PDGFRA (exons 12,14 and 18) were performed on genomic DNA extracted from paraffin-embedded tumour tissue through the commercial QiAamp DNA FFPE Tissue Kit (QIagen) using a combination of polymerase chain reaction (PCR) amplification and automated sequencing.

To exclude other KIT or PDGFRA mutations, PDGFRA gene exons 12, 14, and 18 and KIT gene exons 8, 9, 11, 13, 14, 17, and 18 were sequenced on freshly frozen tumour specimens by the Sanger sequencing method. DNA was isolated by the QIAmp DNA Mini kit (Qiagen, Milan, Italy) in accordance with the manufacturer’s directions. Each exon was amplified with PCR amplification using specific primer pairs designed with Primer Express 3.0 software (Applied Biosystem) to amplify specific exons. Then, the PCR products were purified with the Qiaquick PCR purification kit (Qiagen) and sequenced on both strands using the Big Dye Terminator v1.1 Cycle Sequencing Kit (Applied Biosystems). Sanger sequencing was performed on an ABI 310 Genetic Analyser (Applied Biosystems).

### 3D-Structural comparison and docking analysis

The unique 3D structure of PDGFRA presently available in the Protein Data Bank (PDB) is the auto-inhibited conformation (PDB id 5K5X, resolution = 2.168 Å). To evaluate the possible consequences of the PDGFRA p.His845_Asn848delinsPro variant in relation to the interaction with imatinib, we also considered the structure of c-KIT co-crystalized with imatinib (PDB id 1T46, resolution = 1.6 Å; sequence identity with PDGFRA of 67%). The two proteins were compared at the sequence level with LALIGN and at the structural level with the jCE algorithm, a Java port of the original Combinatorial Extension (CE) algorithm (Implementation by Andreas Prlić)^13^. To evaluate the role of our PDGFRA mutant, Chimera v1.11.2 was used^14^. Then, to demonstrate the inaccessibility of the ATP binding site of the PDGFRA in the auto-inhibited state, docking analyses were performed using AutoDock v4.2.6 and AutoDockTools v1.5.6^12^.

Finally, to understand which are the H-bonds and the contacts in play, we conducted a ligand-receptor interactions analysis using LIGPLOT v 4.5.3 and Chimera, closely examining the geometrical characteristics of our complex structures.

The analysis of the ligand-receptor interactions was performed with LIGPLOT v 4.5.3 and Chimera.

### Ethics approval and consent to participate

This study was approved by the local institutional ethical committee of Azienda Ospedaliero-Universitaria Policlinico S. Orsola-Malpighi (approval number 113/2008/U/Tess, 30 September 2008 approval code, approval date).

## Results

### Immunohistochemistry

**Figure 3:**
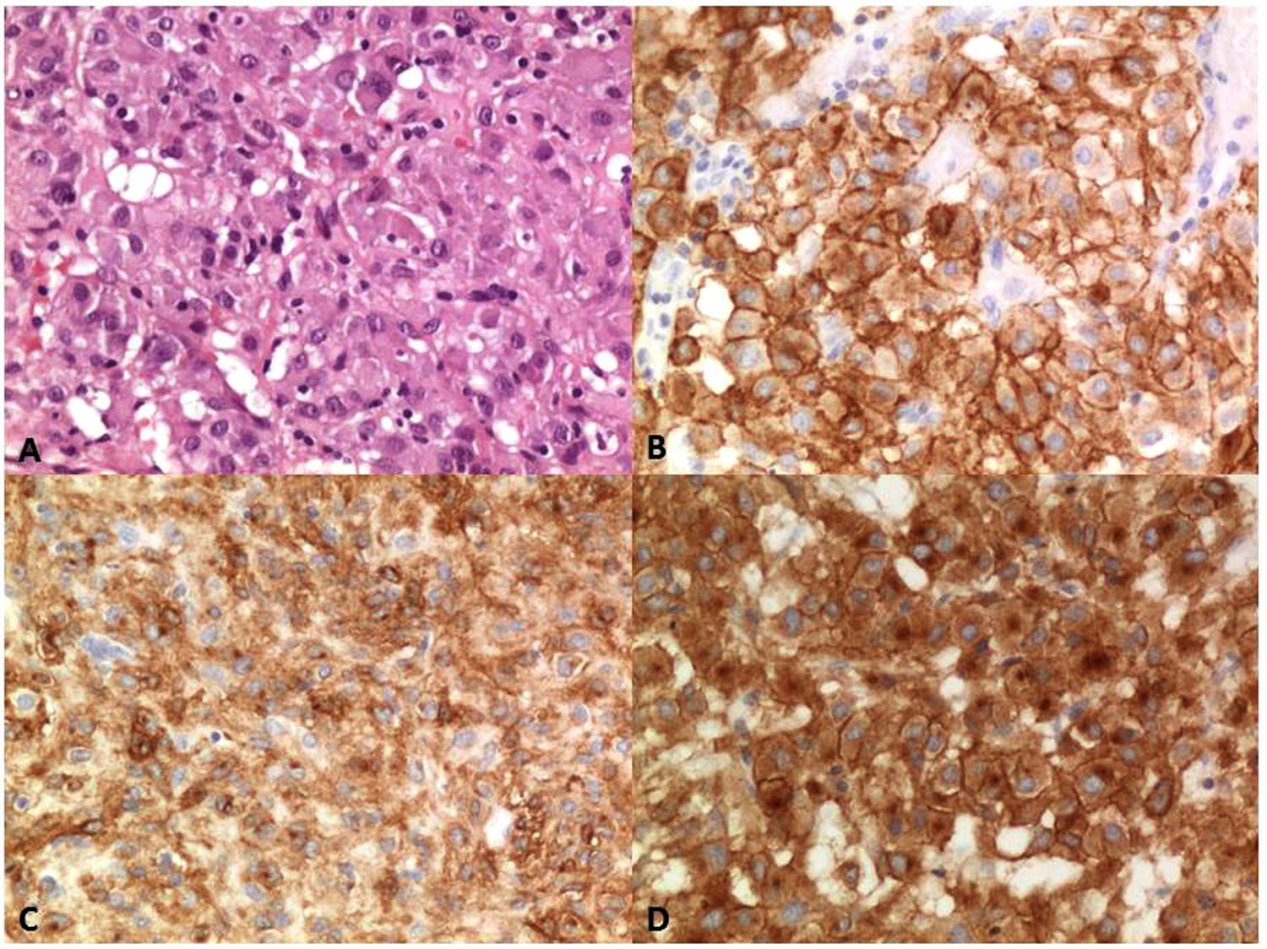
(**A**) Epithelioid morphology; (**B**) diffuse and strong DOG1 staining; (**C**) heterogeneous and patchy CD 117 staining; (**D**) diffuse PFGFRA expression with a membranous and cytoplasmatic staining

The histological examination revealed an epithelioid gastrointestinal stromal tumour comprising polymorphous cells arranged in nests and sheets with eosinophilic cytoplasm, distinct cellular borders and peripherally placed nuclei. There was mild nuclear atypia and no necrosis. The mitotic rate was 4/50 HPF. The immunohistochemistry revealed a strong and diffuse positivity for DOG-1 and a patchy and heterogeneous staining for CD117/c-kit. The tumour cells showed a paranuclear dot-like staining (Golgi pattern) for PDGFRA accompanied by variable cytoplasmic and membranous staining (Figs 2B,C and 3A).

### Molecular analysis

**Figure 4:**
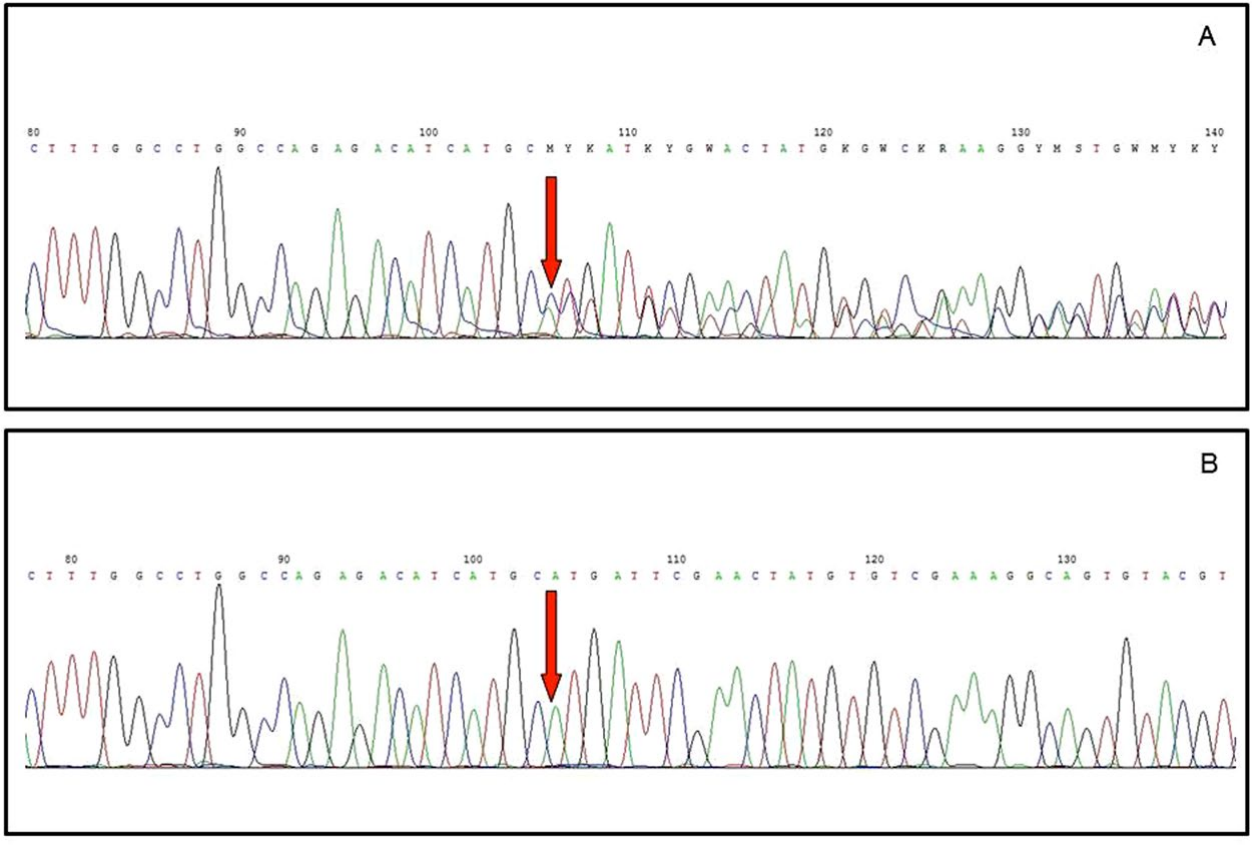
(**A**) Chromatogram showing a deletion of 10 bp and the insertion of a cytosine (c.2534_2543delinsC) affecting exon 18 of PDGFRA (involving residues from 845 to 848: p.His845_Asn848delinsPro); (**B**) chromatogram showing the counterpart wild-type exon 18 of PDGFRA.

A mutation consisting of a deletion of 10 bp and the insertion of a cytosine (c.2534_2543delinsC) affecting exon 18 of PDGFRA (involving residues from 845 to 848: p.His845_Asn848delinsPro) was detected (NM_006206 - GenBank) (Fig. 4). KIT resulted as a wild-type for the hotspot exons examined.

That mutation was confirmed by the Sanger sequencing analysis, and none of the other exons showed any additional alteration.

### 3D-Molecular evaluation

The p.His845_Asn848delinsPro mutant was evaluated adopting the PDB structure 5K5X of PDGFRA in the auto-inhibited conformation (sequence identity is 90%) in which the JM domain overlaps with the kinase active site. We adopted the structure of active form KIT co-crystallized with imatinib (PDB: 1T46, sequence identity 67%) as a reference to determine whether our variant could affect the imatinib binding site.

**Figure 5:**
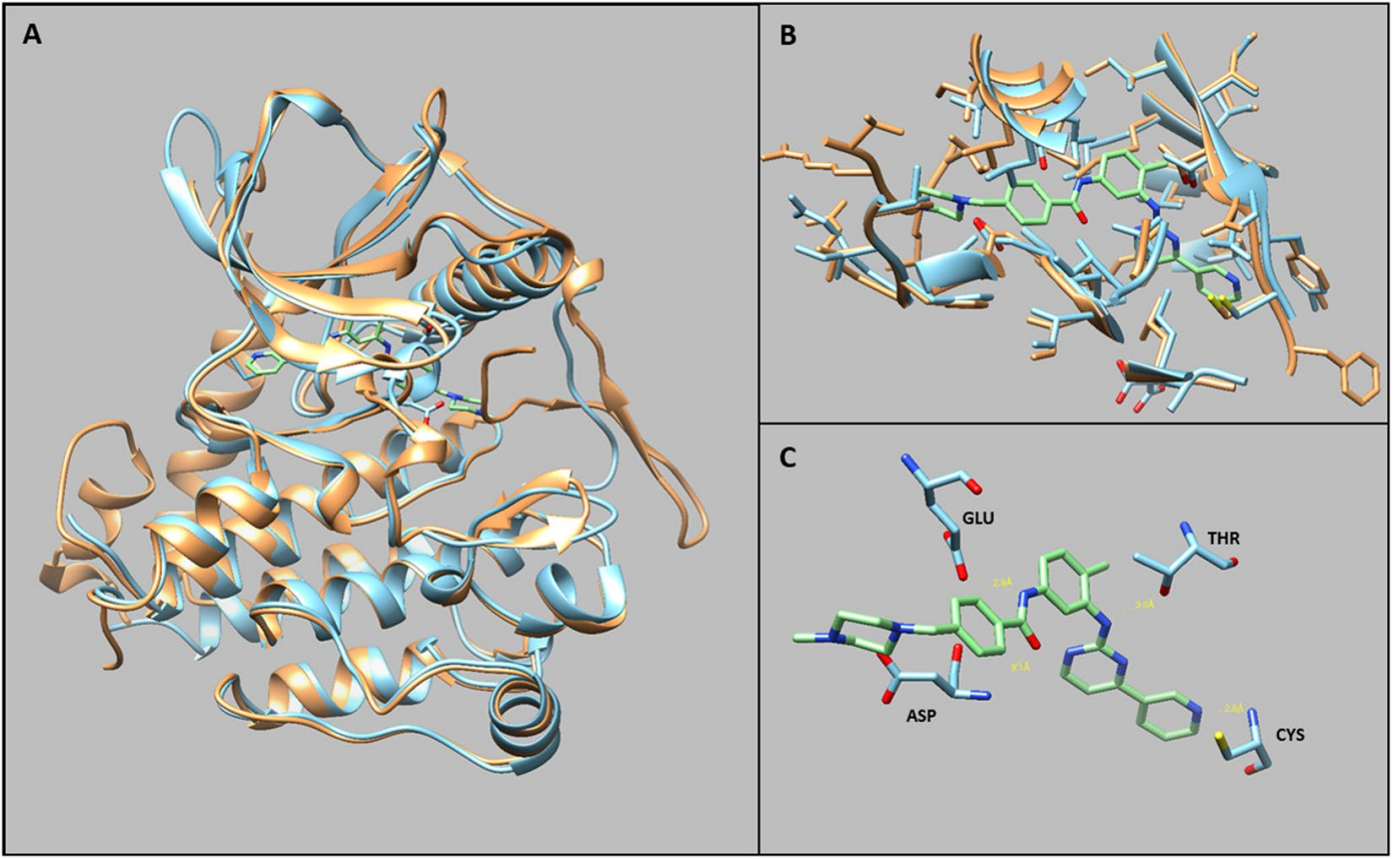
(**A**) Structural superimposition of PDGFRA (PDB:5K5X) and c-Kit co-crystalized with imatinib (PDB: 1T46). (**B**) Structural alignment showing the lateral side chain of the imatinib binding site of PDGFRA and KIT. (**C**) Focus on the h-bonds of the conserved residues involved in the imatinib binding.

**Figure 6:**
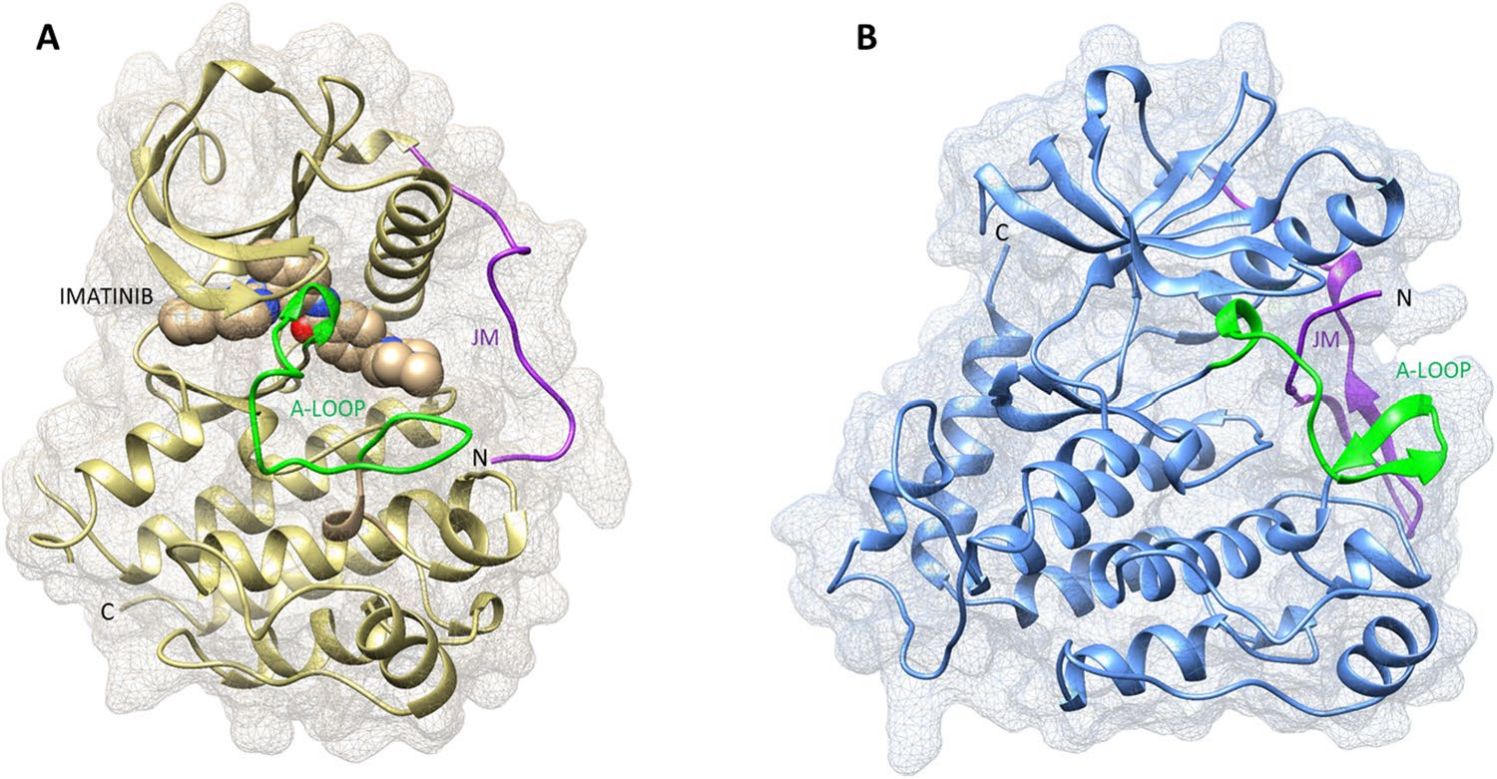
Crystalized structures of c-KIT and PDGFRA. (**A**) c-KIT co-crystalized with imatinib (PDB: 1T46). (**B**) PDGFRA in the auto-inhibited conformation (PDB:5K5X). The activation loop (A-loop) is highlighted in green. Highlighted in purple is the juxta-membrane (JM) domain which in the auto-inhibited PDGFRA shields the kinase active site.

The two structures are superimposable with a RMSD value of 1.39 Å (Fig. 5A). The two sequences share 61% identity and 75% similarity. The docking analysis suggests that the imatinib is stabilized near the ATP binding site only in the active conformation, suggesting that the particular arrangement of the JM domain in the auto-inhibited conformation prevents imatinib binding (Fig. 6A,B).

Our analysis suggests that the binding with imatinib is conserved in the variant. Indeed, the docking simulation shows that PDGFRA p.His845_Asn848delinsPro stabilizes the imatinib binding site with the residues that are conserved in KIT. In particular, the four crucial residues are Glu, Thr, Asp, and Cys (E644, T674, D836, C677 in PDGFRA and E640, T670, D810, and C673 in KIT; Fig. 5C). These residues are in a highly conserved region (Fig. 5B) and not mutated in our variant p.His845_Asn848delinsPro, suggesting that the variant preserves the imatinib binding site.

## Discussion

Herein, we report on a metastatic GIST patient with a mutation of the PDGFRA gene in exon 18 involving the p.His845_Asn848delinsPro residues responding to first-line imatinib. The frequency of this type of mutation is very low, and its sensitivity to imatinib has been previously shown *in vitro* in one case only^1,2^. Thus, in addition to the *in vitro* efficacy data, moving back from the bedside of our case report to the bench, we tried to provide an *in silico* modelling explanation for the sensitivity to imatinib of the p.His845_Asn848delinsPro exon 18 PDGFRA mutation. To this end, we adopted KIT co-crystallized with imatinib as a reference to determine whether our variant could affect the imatinib binding site.

As shown, our computations support the data that imatinib interaction is stabilized by the same lateral chains as in KIT which are conserved in PDGFRA (E644, T674, D836 and C677). On the basis of this assumption, we observed that our variant did not affect these residues; therefore, it is highly likely that it would play no role in activation and that it will not directly affect the binding of imatinib, consistent with the clinical history of the presented case.

To date, the same evidence has been already reported for the PDGFRA exon 18 DIMH842-845, M844_S847del and I843_D846del variants, which have been found to be *in vivo* sensitive to imatinib and for which 3D-modelling has been already built.

However, from a clinical point of view, our report would first highlight the concept that PDGFRA-mutated GIST is an heterogeneous group of diseases with a different spectrum of sensitivity to imatinib. Therefore, it is critical that mutational analyses be performed in as much detail as possible, and, given the rarity of this subgroup of GIST, the annotation of all types of mutations found should be collected. In addition, the modelling investigation of all mutations may constitute a powerful tool to predict imatinib sensitivity, helping clinicians in the decision-making when a mutation not yet functionally investigated is found^15^. In clinical practice, it could be especially useful for high-risk limited disease when an adjuvant treatment should be chosen. Accordingly, a reliable PDGFRA model is mandatory in the future in order to build reproducible and comparable data that are traunsferable into daily practice.

Moreover, molecular modelling may provide new criteria to better define, at a molecular level, the affinity or lack thereof other TKIs to these rare mutations^11^. In particular, it could be interesting to evaluate the affinity to novel compounds currently under development, such as BLU-285 and DCC-2618, which until now have been shown to be particularly promising in PDGFRA D842V mutants; however, nothing is yet known for other PDGFRA mutants in GIST^16,17^.

In the future, a whole molecular characterization of PDGFRA mutants other than D842V, coupled with protein-ligand interaction modelling, could help to build a more representative molecular signature of this rare subgroup of GIST^18^.

## Conclusions

In conclusion, the His845_Asn848delinsPro exon 18 PDGFRA mutation, even if rare, has clinical importance because it is sensitive to imatinib, and that sensitivity, already shown *in vitro*, is likely affected by the conformation of the receptor. Thus, molecular modelling, especially for rare mutations, may represent a reliable tool for the prediction of clinical outcomes and treatment selection.

## Data Availability

All datasets used and/or analysed during the current study are available from the corresponding author upon reasonable request.
